# Thyroid nodules with indeterminate cytology: association between nodule size, histopathological characteristics and clinical outcome in differentiated thyroid carcinomas — a multicenter retrospective cohort study on 761 patients

**DOI:** 10.1007/s13304-021-01096-2

**Published:** 2021-06-07

**Authors:** Federico Cozzani, Dario Bettini, Matteo Rossini, Elena Bonati, Simona Nuzzo, Tommaso Loderer, Giuseppe Pedrazzi, Alberto Zaccaroni, Paolo Del Rio

**Affiliations:** 1 General Surgery Unit, Parma University Hospital, Parma, Italy; 2 Endocrine Surgery Unit, “Morgagni-Pierantoni” Hospital, Forli, Italy; 3 Medicine and Surgery Department, Parma University, Parma, Italy

**Keywords:** Thyroid, FNAC, Guidelines, Differentiated Thyroid Carcinoma, Tailored Surgery, Conservative Surgery

## Abstract

A great number of surgical diagnostic procedures are performed every year for thyroid nodules that are included in undetermined cytological classes that reveal to be malignant thyroid carcinomas in one-third of cases. In the most recent guidelines, lobectomy is the most recommended surgical approach for this classes of nodules, but total thyroidectomy is the recommended treatment for undetermined nodules larger than 4 cm. The main study aim is to support or question the dimensional criteria as an independent clinical decision element for undetermined thyroid nodules management. We examined data regarding 761 patients undergoing thyroid surgery for undetermined thyroid nodules at two high-volume endocrine surgery units in Italy. Patients were divided into three groups based on the preoperative size of the nodules (*N* < 1, 1 < *N* < 4, *N* > 4 cm). Among the patients belonging to the different groups, we analyzed: differences in malignancy rate, histological characteristics of invasiveness and neoplastic aggressiveness, rates of recurrence and response to therapy. Nodule size (evaluated as a categorical variable and as a continuous variable) did not show any statistically significant correlation with the rate of malignancy, histopathological characteristics of tumor aggressiveness and the patient’s clinical outcome. Most of the tumors found were included in the low risk class (79.2%) and only one was classified as high risk. Follow up of cancer cases showed excellent results in terms of survival, response to therapy and disease recurrence. Malignant thyroid tumors of any size resulting from a nodule identified as cytologically indeterminate are usually characterized by a low risk follicular pattern, well-differentiated and with an excellent outcome. As a result, preferring an extended surgical attitude for undetermined nodules based on tumor size, in absence of other risk factors, can lead to overtreatment in a significant percentage of cases.

## Introduction

After the introduction of increasingly refined imaging methods, the incidence of thyroid nodules worldwide is estimated to be between 20 and 60%, with differences related to gender, age and geographical location.

There has been a simultaneous increase in the incidence of thyroid cancer due to a raising number of diagnoses of asymptomatic subclinical carcinomas, secondary to an improvement in diagnostic technology and increased surveillance.

The cytological examination by needle aspiration has become the standard for the evaluation and management of thyroid nodular pathology, as defined by the guidelines of the American Thyroid Association (ATA), in the 2009 and 2015 versions, and confirmed by the main European guidelines [1, 2].

In 2008, the Bethesda System for Reporting Thyroid Cytopathology (BSRTC) was introduced for the cytological evaluation of nodules. The BSRTC defines six diagnostic categories with increasing suspicious characteristics for malignancy: non-diagnostic (Class 1), benign (Class 2), atypia of indeterminate meaning/follicular lesion of indeterminate meaning (Class 3), follicular neoplasm/Hurtle’s neoplasm (Class 4), suspicious for malignancy (Class 5), malignant (Class 6) [3].

In Italian clinical practice is also used the cytological classification of SIAPEC, published in 2007 with five risk classes and revised in 2014 (subdivision into six classes superimposable to BSRTC) [4].

Although the routine use of cytological examination on thyroid nodules has decreased the total number of patients requiring diagnostic surgery, there is still a high percentage of patients, who undergo surgery to obtain a definitive histological diagnosis based on an indeterminate cytology.

The ATA guidelines, published in 2006 and then updated in 2009 and 2015, give clear indications regarding the management of thyroid nodules with indeterminate cytology [1].

The main objective of thyroid surgery for nodules defined as atypia of undetermined significance (AUS) or follicular lesion of undetermined significance (FLUS) or follicular neoplasm (FN) is to establish a histological diagnosis associated with a complete removal of the pathological nodule, in order to perform the best surgical and medical therapy, reducing the risks of excessive surgical extension and related adverse events.

The surgical option (total thyroidectomy vs unilateral lobectomy with possible completion lobectomy) is influenced by several factors which include: risk factors related to a greater risk of malignancy (dimensions greater than 4 cm, neoplastic familiarity, history of radiation exposure), ultrasound features, cytological category and ancillary diagnostic tests (molecular biology). All these risk factors must be further associated with the patient’s preference, the presence of contralateral nodularity, a possible coexisting hyperthyroidism and the presence of patient’s comorbidity [1].

The ATA guidelines 2015 maintain the 4 cm dimensional cut-off as an absolute indication for total thyroidectomy.

A recent study by Valderrabano et al. has attempted to deny this absolute indication, analyzing the clinical and histopathological outcome of indeterminate nodules greater or less than 4 cm. The results did not show statistically significant differences such as to confirm the indication to total thyroidectomy for nodules larger than 4 cm suggested in the ATA guidelines. The American Group therefore brought attention to a need for re-evaluation of the surgical therapy for indeterminate nodules, proposing the extension of diagnostic lobectomy indication for all the nodules belonging to these classes, avoiding the single dimensional criterion as an indicator of greater malignancy or biological aggression [5].

## Materials and methods

### Study project

The present study, multicenter, retrospective, observational, had as its main objective the analysis of the association between the size, measured by ultrasound, of thyroid nodules with indeterminate cytology and histopathological post-operative results (rate of malignancy and tumor aggressiveness criteria).

The centers involved in this study are high volume Italian centers with consequent experience in thyroid cancer surgery:

- General Surgery Unit, Parma University Hospital.
- Endocrine Surgery Unit, AUSL Romagna, “Morgagni-Pierantoni” Hospital, Forlì.

### Study group

Seven hundred and sixty-one patients who underwent thyroid surgery with indeterminate cytology nodules in the period between January 2010 and December 2018. All nodules included in the study were subjected to cytological examination after the analysis of ultrasound characteristics and medical history risk criteria.

The exclusion criteria were: evidence of malignancy, previous chordal paresis, metastatic lymph nodes (local or distant) diagnosed by US, CT and FNAC during the preoperative study phase, or preoperative levels of serum calcitonin > 100 pg/ml.

We analyzed patients age, gender, date of surgery (pre/post ATA 2015 guidelines introduction), type of surgery performed (lobectomy vs total thyroidectomy), cytological diagnosis (follicular proliferation not better classified because diagnosed in a period before the BSRTC introduction (FP) vs Class 3 of BSRTC vs Class 4 of BSRTC) or (Class 3 vs Class 3a vs Class 3b of SIAPEC classification), nodule size found during pre-operative tests, multinodularity (presence of contralateral nodules), definitive histological report, malignancy criteria present in case of differentiated carcinoma (DTC), degree of response to therapy, eventual finding of disease recurrence (follow-up at least 6 months). The superimposability of the cytological classifications allowed us to standardize the data of the two operating units involved in the study. The SIAPEC 3A Class group has been conformed to the BSRTC Class 3 group and the group of nodules classified as SIAPEC Class 3B to the BSRTC class 4. For organizational reasons, it was not possible to obtain a review of all 761 cytological samples by a single examiner, but considering the experience of the two centers involved, that are two high-volume centers, we did not believe that this represented an alteration in the study data. We think that is important to underline that the study is about analysis of clinical and anatomopathological outcome following surgery for indeterminate thyroid nodules, regardless of the cytological subclass.

The nodules with indeterminate cytology not included in either of the two subclasses, diagnosed in a period before the introduction of the new classification, were inserted in a third group called FP (follicular proliferations not better classified). It was not possible to identify an undetermined cytological subclass (Class 3 or Class 4 of BSRTC) for these nodules, but we think that this fact is not influencing the study main aim. They were diagnosed as undetermined nodules and then included in the study even if not belonging to a specific undetermined subclass.

The study population was divided into groups dependent on preoperative size of the thyroid nodules:

- Group 1: nodules less than or equal to 1 cm.
- Group 2: nodules between 1.1 and 4 cm.
- Group 3: nodules greater than 4 cm.

The DTC were divided into three groups:

- Low-risk DTC: (all of the following criteria must be present) intrathyroidal extension, negative resection margins, lymphovascular invasion (< 4 foci for follicular carcinomas), clinical N0 or 5 or less lymph node metastases less than 2 mm, not distant metastasis.
- High-risk DTCs (at least one of the following criteria must be present): massive extrathyroidal invasion (T4), N1b or any other lymph node metastasis greater than 1 cm and/or distant metastasis.
- Intermediate-risk DTCs : remaining cases.

Response to therapy was assessed as recommended by the ATA guidelines for malignant tumors treated with radioactive iodine. DTC group patients with at least 6 months follow-up have been investigated for nodal recurrence and response to therapy assessing the absence of new tumor foci greater than 1 cm.

Differences between dimensional groups were mainly analyzed in: incidence of thyroid carcinoma (malignancy rate), detection rates of histological characteristics of invasiveness, clinical neoplastic aggression and rates of recurrence and response to therapy.

### Study purpose

The ultimate goal is to support or question the dimensional criteria as an independent clinical decision element for indeterminate nodules management. This project is part of a “tailored surgery approach” which has the aim of making conservative surgery for indeterminate thyroid nodules more and more widespread so as to reduce the number of overtreatment and the risk of surgical adverse events.

### Statistical analysis

Comparisons are made between the groups formed on the nodule size measured during preoperative ultrasound examination. Fisher’s exact test and Pearson’s Chi-squared test were used for the categorical variables. Other analysis were performed considering the size of the nodule as a continuous variable. We used Student’s *t* test, Welch’s test, and Mann–Whitney *U* test. The Fisher’s exact test and the Pearson’s Chi-squared test were used to evaluate the association between size and malignancy rate, and the association between nodule size and carcinoma aggressiveness (high, medium and low risk).

## Results

### Population characteristics

Seven hundred and sixty-one patients with an undetermined thyroid nodule diagnosis were included in the study (426 treated at the OU of the General Surgical Clinic of the AOU of Parma and 335 from the database of the Endocrine Surgery Operative Unit of the Hospital “Morgagni-Pierantoni” of Forlì) Table 1. The study population was composed of 591 females (77.7%) and 170 males (22.3%), equally distributed between the two operative units databases. Patients age (average 52.86 years, minimum 18 and maximum 83), showed no difference between the two sites. The population was then divided in two groups: first group including patients under 55 years of age (413 pts—54.3%) and second group including patients older than 55 years (348 pts—45.7%).

**Table 1:** Study group population characteristics

Sample features	Parma (*N* = 426)	Forli (*N* = 335)	Total (*N* = 761)	*p* value
Gender				
Female	322 (76%)	269 (80%)	591 (77.3%)	ns
Male	104 (22%)	66 (20%)	170 (22.7%)	
Age (years)				
Average ± SD	53.9 (± 13.23)	51.4 (± 14.04)	52.86 (± 13.83)	ns
Range	19–83	18–81	18–83	
Age groups				
< 55 aa	217 (51%)	196 (59%)	413 (54.27%)	ns
> 55 aa	209 (49%)	139 (51%)	348 (45.73%)	
Multinodular pathology				
Yes	247 (58%)	117 (35%)	364 (48%)	< 0.0001
No	179 (42%)	218 (65%)	397 (52%)	
Node dimensions (mm)				
Average	20.23 (± 11.67)	25.44 (± 13.19)	22.52 (± 12.62)	
Range	6–80	5–90	5–90	
Dimensional groups				
*N* < 1	69 (16.2%)	23 (6.9%)	92 (12.1%)	ns
1 < *N* < 4	318 (74.6%)	263 (78.5%)	581 (76.3%)	
*N* > 4	39 (9.2%)	49 (14.6%)	88 (11.6%)	
Cytological diagnosis				
Prol. Folic.			191 (25.1%)	–
Thyr 3			184 (24.2%)	
Thyr 4			386 (50.7%)	

At the time of diagnosis 364 patients (47.8%) showed a multinodular thyroid pathology; 397 showed a single nodule found on pre-operative ultrasound. In Parma experience we highlighted a higher incidence of multinodular pathology than Forlì (58% vs 35%).

This difference can perhaps be explained by the characteristic iodine deficiency identified in the past few years in the Parmesan area [6].

The average size of examined nodules was 22.52 mm (5–90 mm).

The population was divided into three groups: the first including nodules smaller than 1 cm in size (92 pts–12.1%); the second including nodules ranging from 1 to 4 cm in size (581 pts–76.3%); the third group with an undetermined thyroid nodule diagnosis larger than 4 cm (88 pts–11.6%).

184 (24.2%) patients underwent surgery with BSRTC Class 3 cytological diagnosis, 386 (50.7%) with BSRTC Class 4 diagnosis and the remaining 191 (25.1%), were treated before BSRTC. These last patients were inserted in a third group defined generically as indeterminate follicular proliferations (FP group).

Considering the total study period, Total thyroidectomy was preferred to a conservative approach; 439 total thyroidectomies (57.7%) and 322 lobectomies (42.3%) were performed as first intervention following diagnosis of thyroid nodules with indeterminate cytology.

After 2015 ATA guidelines introduction, lobectomy rate, that was 34.5% of all surgeries performed in the two centers before this period (2010–2014), raised to 51.3% considering the 355 surgical procedures performed between 2015 and 2018.

This result highlights the indications to treat change introduced after 2015 ATA Guidelines compared to previous surgical indications in 2009 version.

Among all 322 lobectomies performed only 37 totalizations were performed (11.5%) (Table 2).

**Table 2:** Surgical approach

Surgical approach	Total (*N* = 761)
Surgery performed	
Total thyroidectomy	439 (57.7%)
Lobectomy	332 (42.3%)
Rate of totalisations (332 lobectomies performed)	37 (11.5%)
Lobectomy rate	
ATA 2009 GL	140/406 (34.5%)
ATA 2015 GL	182/355 (51.3%)
Rate of totalisations per DTC (71 cases of DTC after lobectomy)	35/71 (49.3%)

Considering all 761 nodules undergoing surgery only 194 were found to be DTC (25.5%) (Tables 3, 4).

**Table 3:** Correlation between malignancy and analyzed variables

Correlations between malignancy and analyzed variables	DTC		*p* value
	Yes (*N* = 193)	No (*N* = 567)	
Gender			
Female	146 (24.7%)	445 (75.3%)	*ns*
Male	47 (27.6%)	123 (72.4%)	
Age groups			
< 55 aa	119 (28.8%)	294 (71.2%)	0.017
> 55 aa	74 (21.3%)	274 (78.7%)	
Dimensional groups			
*N* < 1	18 (19.6%)	74 (80.4%)	0.38
1 < *N* < 4	153 (26.3)	428 (73.7%)	
*N* > 4	22 (25%)	66 (75%)	
Nodules < 1 cm			
Yes	18 (19.6%)	74 (80.4%)	0.173
No	175 (26.2%)	494 (73.8%)	
Nodules between 1 and 4 cm			
Yes	153 (26.3)	428 (73.7%)	0.268
No	40 (22.2%)	140 (77.8%)	
Nodules > 4 cm			
Yes	22 (25%)	66 (75%)	0.934
No	171 (25.4%)	502 (74.6%)	
Cytological diagnosis			
PF group	59/191 (30.9%)		
Class 3 of BSRTC	34/184(18.5%)		
Class 4 of BSRTC	101/386 (26.2%)

**Table 4:** DTC histotypes

Histotypes of DTC	Number of cases	%
Papillary carcinoma (classic var.)	49	25.26
Papillary carcinoma (follicular var.)	80	41.24
Papillary carcinoma (oxyphilic var.)	7	3.61
Hurtle carcinoma	11	5.67
Follicular carcinoma	47	24.23

Within the FP group, the detected malignancy rate was 30.9% (59 DTC out of 191 nodules), in Thyr 3 group 34 DTCs were detected out of 184 nodules examined (18.5%) and the group including Thyr 4 nodules showed a malignancy rate of 26.2% (101 DTC out of 386 nodules examined).

A separate analysis did not reveal statistically significant differences between the results of Parma and Forlì Operative Unit. Malignancy rate was 28.4% in Forlì Endocrine Surgery Unit experience and 23% in Parma General Surgery Unit.

The malignancy rates of Parma were 25% for PF group, 18% for BSRTC Class 3 group and 25.2% for BSRTC Class 4 group; in Forlì were 32.9% for PF group, 19.6% BSRTC Class 3 group and 27.9% in BSRTC Class 4 group. No statistically significant differences in malignancy rate were showed (*p* = 0.38) (Table 3).

In the group of nodules smaller than 1 cm malignancy rate was 19.6% (18 DTC out of 92 nodules); in the group with nodules between 1 and 4 cm the recorded rate was 26.3% (153 DTC out of 581 nodules). In conclusion, the group of nodules with a diameter bigger than 4 cm was analyzed and also in these patients the size did not show a significant effect on malignancy rate corresponding to 25% (22 DTC on 88 nodules). The same result was also detected by dividing the population between the two hospitals.

Among the variables analyzed, the only one that showed a significant effect on malignancy rate was age of less than 55 years. A malignancy rate of 29% was found in the younger group (119 out of 413), significantly higher than the older age group which showed a 21.3% rate (74 out of 348 patients) (*p* = 0.017). Female gender showed a greater association with DTC only within the group of subjects under the age of 55 years (*p* = 0.011).

The rate of completion thyroidectomy following lobectomy, performed because of histological diagnosis of malignancy, showed no difference between the three dimensional groups (*p* = 0.303).

### Cancer aggressiveness (Tables 5, 6)

**Table 5:** DTC risk classes

Cancer aggressiveness	*N* < 1 cm (*N* = 18)	1 cm < *N* < 4 cm (*n* = 153)	*N* > 4 cm (*N* = 22)	Total DTC (*N* = 183)	% total nodules (*N* = 761)
Low-risk DTC	17/18 (94.4%)	113/153 (73.8%)	15/22 (68.2%)	145/183 (79.2%)	145/761 19.05%
Intermediate-risk DTC	1/18 (5.6%)	29/153 (18.95%)	7/22 (31.8%)	37/183 (20.2%)	37/761 (4.86%)
High-risk DTC	–	1/143 (0.65%)	–	1/183 (0.55%)	1/761 (0.13%)

**Table 6:** Correlation between nodule size tumor aggressiveness and differentiated thyroid cancer patients with a previous indeterminate (Thy 3) cytology have a better prognosis than those with suspicious or malignant FNAC reports

Cancer aggressiveness	*N* < 1 cm (*N* = 18)	1 cm < *N* < 4 cm (*n* = 143)	*N* > 4 cm (*N* = 88)	Total (*N* = 183)
Extracapsular/lymphovascular invasion	1/18 (5.6%)	29/143 (20.3%)	7/22 (31.8%)	37/183 (20.2%)

Among malignant nodules on post-operative histological examination, we analyzed the anatomopathological characteristics of malignancy, looking for a correlation between nodule size and extracapsular or lymphovascular invasion.

As regards the extracapsular or lymphovascular invasion, we found an incidence of 6% in DTC group < 1 cm, 20.3% (29 out of 143) in DTC group between 1 and 4 cm and 31.8% (seven out of 22) in those larger than 4 cm; statistical analysis showed no statistically significant differences (*p* = 0.120) (Tables 5, 6).

Comparing only the group of DTCs larger than 4 cm with others we showed an incidence of extracapsular invasion of 31.8% against 18.6% (30 out of 161 DTC) (*p* = 0.149). Only seven cases of lymph node metastases were recorded, six were located at the 5th level (N1a) while just one case was at the laterocervical level (N1b). In only one case lymph node metastases was detected deriving from an indeterminate nodule larger than 4 cm. The remaining six cases (five N1a and one N1b) were found in patients related to the group with indeterminate nodules of sizes between 1 and 4 cm.

The percentage of patients who underwent radiometabolic therapy after surgery was subsequently analyzed. We collected data about 148 patients and only 56.7% (84 out of 148) showed the necessity of a post-operative treatment. No statistically significant difference were identified between the three groups (*p* = 0.221).

We also analyzed the follow up data of 129 patients diagnosed with DTC (average 49.35 months with a minimum of 6 months and a maximum of 109). Only seven remote lymph node recurrences were found on 127 patients followed (5.5%), with no statistically significant difference between 5% of the DTC group > 4 cm and 7% of the rest of the population (*p* = 0.835). Incomplete response to therapy only in five cases out of 126 (4%) without significant differences between the DTC group > 4 cm and the remaining smaller carcinomas (*p* = 0.402) There were no cases of death related to the DTCs involved in the study.

## Discussion

The analysis of results revealed an absence of significant statistically correlation between the size of thyroid nodule with indeterminate cytology and the rate of malignancy, characteristics of tumor aggression and oncological outcome in the event of DTC; confirming the results of the American study group [5].

Only 11.6% of all nodules included in the study have been diagnosed as larger than 4 cm.

This data is not surprising because of the massive execution of ultrasound examinations, which are also performed under screening, in our areas due to the very high incidence of thyroid nodular pathology. This greatly reduces the number of nodules that escape clinical and ultrasound surveillance, thus not being able to reach large dimensions without undergoing further early diagnostic procedures.

The presence of extracapsular or lymphovascular invasion and the evaluation of cancer aggressiveness is not statistically significant but we highlighted an increase in malignancy rate with an increase in nodule diameter (6% vs 20.3% vs 31.8%). This result can be due to the low number of patient belonging to malignant nodules > 4 cm group, but only further prospective and larger studies can give us stronger answers.

Most of DTCs found showed characteristics that can be included in the low risk category according to 2015 ATA guidelines, a minority were included in the intermediate risk category (20.2% of DTCs, corresponding to 4.73% of undetermined nodule cases included in the study) and only 1 (0.13% of the total sample) was included in the high risk category for the detection of stage laterocervical metastases (N1b). The present study showed that most malignant tumors resulting from nodules with indeterminate cytology are low risk lesions with follicular appearance patterns [7–10].

Different from many other studies result is the non-correlation between female or male gender and a greater rate of malignancy. In our study population we found a statistically significant correlation between the age of less than 55 years and malignancy rate. Female gender seems to play a role favoring the development of DTC only within the younger population; this difference disappears in the group of patients over 55 years.

We found a completion thyroidectomy rate of 11% following a conservative approach.

We must remember that total thyroidectomy is no longer recommended for all DTCs larger than 1 cm. The 2015 ATA Guidelines suggested that lobectomy can be an initial treatment sufficient for DTCs smaller than 4 cm, NIFTP of any size, minimally invasive follicular carcinomas and papillary carcinomas variant follicular encapsulated and intrathyroidal [1, 11].

This reduction in surgical aggression in DTC treatment is supported by two evidences: the extension of surgery does not seem to have an effect on survival or recurrence rate [12, 13] and the complication rate of thyroid lobectomies is different from total thyroidectomies [14].

A more conservative resection allows to preserve functionality in 80% of cases [15], and the outcome and complication rate of two-stage thyroidectomy are similar to those of total thyroidectomy performed in a single time [16].

In this study we found no association between tumor size and carcinoma aggressiveness. Based on histopathological data, a lobectomy would probably have been the most suitable initial treatment for most of the patients included in this study.

## Conclusion

Malignant thyroid tumors of any size resulting from a nodule identified as cytologically indeterminate are usually characterized by a low risk follicular pattern, well-differentiated and with an excellent outcome. As a result, preferring an extended surgical attitude for undetermined nodules based on tumor size can lead to overtreatment in a significant percentage of cases, exposing the patient to unnecessary surgical risks and complications, and the need to take life-long hormone replacement therapy.

The present study, therefore, suggests that a therapeutic or diagnostic lobectomy should be the treatment of choice for most thyroid nodules with indeterminate cytology, in the absence of other indications for total thyroidectomy, regardless of the size of the nodule.

Further prospective multicenter studies are needed to strengthen the conclusions and consequent therapeutic hypotheses of the present retrospective study.

## Data Availability

The datasets used and/or analyzed during the current study are available from the corresponding author on reasonable request.
